# Physiological alterations after a marathon in the first 90-year-old male finisher: case study

**DOI:** 10.1186/2193-1801-3-608

**Published:** 2014-10-17

**Authors:** Sandro Manuel Mueller, Beat Knechtle, Patrizia Knechtle, Marco Toigo

**Affiliations:** Exercise Physiology Lab, Institute of Human Movement Sciences, ETH Zurich, Winterthurerstrasse 190, 8057 Zurich, Switzerland; Institute of General Practice and Health Services Research, University of Zurich, Pestalozzistrasse 24, 8091 Zurich, Switzerland

**Keywords:** Dual-energy X-ray absorptiometry, Peripheral quantitative computed tomography, Countermovement jump, Multiple one-legged hopping, Impulse

## Abstract

**Introduction:**

Endurance performance decreases during ageing due to alterations in physiological characteristics, energy stores, and psychological factors. To investigate alterations in physiological characteristics and body composition of elderly master athletes in response to an extreme endurance event, we present the case of the first ninety-year-old official male marathon finisher.

**Case description:**

Before and directly after the marathon, a treadmill incremental test, dual-energy X-ray absorptiometry, peripheral quantitative computed tomography, mechanography, and dynamometry measurements were conducted. The athlete finished the marathon in 6 h 48 min 55 s, which corresponds to an average competition speed of 6.19 km h^-1^.

**Discussion and Evaluation:**

Before the marathon,  was 31.5 ml min^-1^ kg^-1^ body mass and peak heart rate was 140 beats min^-1^. Total fat mass increased in the final preparation phase (+3.4%), while leg fat mass and leg lean mass were slightly reduced after the marathon (-3.7 and -1.6%, respectively). Countermovement jump (CMJ) peak power and peak velocity decreased after the marathon (-16.5 and -14.7%, respectively). Total impulse during CMJ and energy cost of running were not altered by the marathon. In the left leg, maximal voluntary ground reaction force (*F*_m1LH_) and maximal isometric voluntary torque (MIVT) were impaired after the marathon (-12.2 and -14.5%, respectively).

**Conclusions:**

Side differences in *F*_m1LH_ and MIVT could be attributed to the distinct non-symmetrical running pattern of the athlete. Similarities in alterations in leg composition and CMJ performance existed between the nonagenarian athlete and young marathon runners. In contrast, alterations in total body composition and m1LH performance were markedly different in the nonagenarian athlete when compared to his younger counterparts.

## Background

It is scientifically well established that endurance performance decreases with aging. Even well-trained master athletes suffer from reductions in several physiological variables. These variables can be separated into central and peripheral factors undergoing age-related decreases. The central alterations include at least three factors. First, maximal heart rate is reduced irrespective of activity level (Tanaka *et al.*^2001^). Second, elderly athletes have a smaller stroke volume in relation to young athletes (Rivera *et al.*^1989^) presumably because of an increased peripheral resistance (Saltin 1986). Third, a reduced maximal expiratory ventilation volume occurs because of an impaired elasticity of the rib cage and lung tissue. The peripheral factors consist mainly of a reduced blood flow to the legs (Donato *et al.*^2006^, Wahren *et al.*^1974^) and a decline in muscle fiber size as well as contractile performance (Aagaard *et al.*^2010^). The reduction in leg blood flow relies on an increased peripheral resistance that is based on a decreased elasticity of the arteries and arterioles (Franklin *et al.*^1997^). A further peripheral factor that might be decreased with aging is oxidative enzyme activity. Although enzyme activity (*e.g.* succinate dehydrogenase) is hardly changed with increasing age in sedentary men (Suominen *et al.*^1977^), the decreased training volume and intensity in master athletes might be responsible for a decrease in succinate dehydrogenase activity in this population group (Trappe *et al.*^1995^). Taken together, all these mentioned reductions can lead to a lower  (Kusy and Zielinski 2014, Pollock *et al.*^1997^) and decreased endurance performance (Wiswell *et al.*^2001^) in master athletes relative to young athletes.

**Figure 1:**
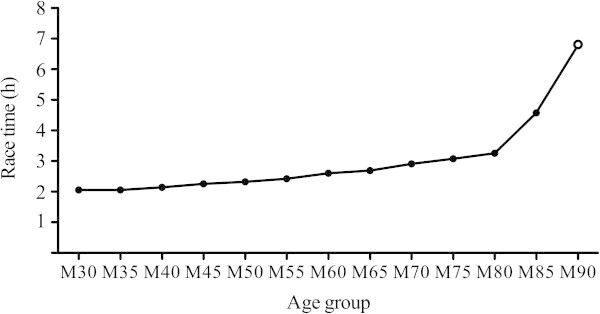
**Marathon world best times per age group.** Full dots, world best times per age group; open dot, marathon-finishing time for present athlete. Data was obtained from the record lists of the International Association of Athletics Federations and World Master Athletics.

The described alterations in physiological characteristics in master athletes lead to performance decrements, which in turn result in progressively greater race times (Figure 1). For each age group of athletes between the age of 30 (M30) up to 69 (M65) years, the best race time increases by ~5% per age category when compared to the actual marathon world record (2 h 03 min 23 s, Figure 1). From M65 to M80, the best time increases by 10% per age group (Figure 1). Therefore, the race time of 80 years old master athletes is ~60% greater than for 30 years old athletes. For M85, a disproportional increase in race time can be observed (Figure 1). In fact, the best time of M85 is ~120% greater than M30. Based on this progression, one might hypothesize that there are far-reaching physiological alterations occurring at the age of 70 and 85 years (*i.e.* senescence). Interestingly, up to date, there is no official world best time for men older than 90 years.

The marked increases in world best times with increasing age partly rely on alterations in the above-described physiological variables of the master athletes. These mentioned alterations in physiological variables accompany decreases in neuromuscular function [*e.g.* peak jumping power (Michaelis *et al.*^2008^) and metabolic power (Rittweger *et al.*^2009^)], which might further negatively affect performance. In addition, energy expenditure during the competition is different between young and older marathon runners, because carbohydrate availability as well as glycogen stores are lower in the elderly (Meredith *et al.*^1989^). Hence, endurance performance in the master athlete is affected by the alterations in several physiological variables, neuromuscular performance, as well as energy expenditure, whereby measuring body composition can approximate the latter. To investigate alterations in physiological variables, neuromuscular performance, and body composition of elderly master athletes in response to an endurance event, it is necessary to measure these variables before and directly after the event. Up to date, there are no studies, which investigated acute alterations in body composition and neuromuscular fatigue in very old master athletes before and after an endurance event.

In the present study, we report the case of the first ninety-year-old man officially completing a marathon. Before and after the marathon, an incremental treadmill test was performed to assess cardiovascular performance. Furthermore, mechanography and dynamometry measurements were conducted to assess neuromuscular function. Finally, to investigate whether the marathon would make an impact on body composition, bone, fat and lean tissue variables were measured by dual-energy x-ray absorptiometry (DXA) and peripheral quantitative computed tomography (pQCT).

## Methods

### Participant

The participant was a ninety-year old male master athlete. As described in detail previously (Knechtle *et al.*^2010^), he started with regular training at 64 years of age. His training routine consisted of three runs per week, with each run lasting ~1 h. Two training sessions were of low intensity (~75% peak heart rate), while the third training session was of moderate intensity (~85% peak heart rate). One month before the marathon, a thorough cardiac examination was performed in order to exclude a potential coronary heart disease. Echocardiography revealed a normal left ventricular function, a 24-hour electrocardiogram (ECG) showed no cardiac arrhythmia and in the exercise ECG, the runner achieved 112% of the age-, gender-, body height-, and body weight-estimated target with no alterations in the ST-segment. The final preparation for the marathon (week 6 to week 3 prior to the marathon) was impeded by pain in the left foot (first metatarsophalangeal joint), whereby only a minimal training volume could be maintained for three weeks. MRI scans showed no fracture and no lesion of tendons. All procedures applied in the study were approved by the Institutional Review Board of Kanton St. Gallen and the athlete gave his written informed consent. Additional informed consent was obtained from the participant for whom identifying information is included in this article.

### Pre-race preparation and race

The athlete started at the ‘Swiss City Marathon’ in Lucerne on October 27, 2013, just 5 d after his 90th birthday. During the race, he was supported by his medical doctor who also trained with him during the final preparation phase. During the preparation phase, the athlete completed a training session over the full marathon distance to prepare race tactics as well as fluid and food intake. The athlete completed this training marathon in 6 h and 6 min, which corresponds to an average velocity of 6.92 km h^-1^. During the competition, food and drinks (*i.e.* energy bars, carbohydrate gels, hypotonic and caffeinated drinks) were transported and served by his support. The supporter recorded all food and fluid intakes accordingly. The ambient air temperature was 9°C at the start of the marathon while heavy rain was falling. After the first half of the marathon, rainfall decreased and the temperature increased to 14°C by the end of the race.

### Experimental overview

The participant reported to the laboratory for the first time 5 d before the marathon. He was advised to arrive well nourished (*i.e.* with filled glycogen stores) and to follow exactly the same preparative routine as planned for the day of the marathon (to simulate the immediate pre-competition situation). Directly after the race, the participant returned to the laboratory for the second testing session. During the transfer from the field to the laboratory, the athlete was mainly sitting, but short intermittent walking periods were applied. Nutritional and fluid intakes were not allowed until DXA and pQCT measurements were completed. Prior to the DXA measurement, the athlete rested for 5 min in a supine position. All measurements were completed within 3.5 h after arrival at the finish line.

### Measurements

#### Dual-energy X-ray absorptiometry (DXA)

A densitometer (Lunar iDXA™, GE Healthcare, Madison, WI, USA) was used for the determination of body composition as well as bone mineral density (BMD) of the femur and lumbar spine according to the manufacturer’s specifications. The delineation in regions of interest (ROI) for the whole legs was done automatically by the integrated software (enCORE, GE Healthcare, Madison, WI, USA; version 14.10.022). The upper leg ROI were defined manually as follows: ROI upper boundary = horizontal line just below the ischium, ROI lower boundary = horizontal line between femur and tibia, ROI lateral boundaries = outer leg cuts, while the lower leg ROI were defined as follows: upper boundary = horizontal line between femur and tibia; lower boundary = horizontal line through the tibio-talar joint; lateral boundaries = outer lower leg cuts.

#### Peripheral quantitative computed tomography (pQCT)

We measured volumetric bone mineral content (vBMC), volumetric bone mineral density (vBMD), and architecture of the bone at 4, 14, 38 and 66% of the tibia length in both legs as well as muscle density at 66% of tibia length with an XCT 3000 Scanner (Stratec, Pforzheim, Germany) as previously described (Anliker *et al.*^2011^).

#### Jumping mechanography

Three vertical countermovement jumps (CMJ) with freely moving arms (separated by 30 s of rest) were performed on a strain gauge ground reaction force platform (Leonardo Mechanograph®, Novotec, Pforzheim, Germany) linked to a desktop computer using an integrated analog digital board and software system (Leonardo Mechanography GRFP version 4.4, Novotec, Pforzheim, Germany). The participant was instructed to jump as high as possible while keeping his head still during the flight of the jump, and to stand still with arms hanging loosely after landing. After the CMJs and 1 min rest, the participant performed three vertical squat jumps (SJ) with the hands held in place on the hips (separated by 30 s of rest). For both CMJ and SJ, peak power was calculated from the product of force and velocity. From the three valid attempts each (CMJ and SJ), the jump with the highest jump height was used for further analyses. For the impulse analysis, baseline force was determined while the participants stood still on the ground reaction force plate for 1 s. During the CMJ, the measured ground reaction force intersected three times with this baseline value resulting in three areas under the force curve. The three individual impulses (I_1_, I_2_, I_3_) were calculated correspondingly as the areas between the progression of the ground reaction force curve and the baseline force. Finally, maximum voluntary ground reaction force (*F*_m1LH_) was determined by multiple one-legged hopping (m1LH) as previously described (Anliker *et al.*^2011^, Anliker and Toigo 2012). Any jumps with heel contact were excluded from the analysis. Heel contact was controlled visually during the jumping maneuver. *F*_m1LH_ corresponded to peak force during m1LH.

#### Knee extension torque

Knee extensor maximal isometric voluntary torque (MIVT) was tested using a commercially available dynamometer (Con-Trex® MJ, Physiomed Elektromedizin, Schnaittach/Laipersdorf, Germany). After the body of the participant was stabilized with straps and handles, the participant performed three maximal knee extensions separated by 1 min to assess MIVT. Therefore, the lever arm of the dynamometer was fixed at a knee angle of 110° (full extension =180°) and the participant was advised to extend his leg as forcefully as possible.

#### Treadmill step test

The treadmill test was performed on a Pulsar 3p® treadmill (h/p/cosmos sports & medical gmbh, Traunstein, Germany) starting at 4 km h^-1^. Thereafter, the speed was increased every 2 min by 1 km h^-1^. The participant ran until volitional exhaustion. During the treadmill test, the participant was equipped with a facemask (Hans Rudolph, Shawnee, KS, USA). The facemask was connected with an anti-bacterial filter (PALL PRO1087, Pall, East Hills, NY, USA) to an Innocor™ device (Innocor™, Innovision, Odense, Denmark), whereby pulmonary gas exchange and ventilation were continuously measured breath by breath.

### Data analysis

The statistical analysis for each parameter was performed based on the least significant change (LSC). A significant change was assumed if the change in a parameter was higher than LSC [LSC = 1.5 × typical error expressed as a coefficient of variation (CV; Hopkins 2000)]. For all the pQCT and DXA parameters, we used the CV’s determined in our laboratory. CV calculated as the ratio of the standard deviation and the mean for *F*_m1LH_ and peak jumping power correspond to 4.3% and 5.5% (Veilleux and Rauch 2010). Energy cost of running was calculated retrospectively according to the model of di Prampero *et al.* ( 1986):
1

where *C* represents the energy cost of running, *F* is the fraction of  maintained during running at competition velocity on a treadmill, and *v* is the average speed during the competition.

## Results

### Physiological characteristics and race performance

The physiological characteristics of our participant are summarized in Table 1. The participant finished the marathon in 6 h 48 min 55 s (6.19 km h^-1^). He passed the half marathon in 2 h 50 min 47 s (7.41 km h^-1^) but had to reduce his running speed thereafter leading to a second half marathon time of 3 h 58 min 08 s (5.32 km h^-1^). During the first half of the marathon, calculated average  was 24.2 ml min^-1^ kg^-1^ body mass ( ), which dropped to 17.4 ml min^-1^ kg^-1^ body mass ( ) during the second half of the marathon. During the race, he consumed a total of 1.8 l of fluids and 250 g of carbohydrates. While fluid and energy intake were very regular during the first half of the race, the intake became irregular in the second half due to gastrointestinal problems.

**Table 1:** **Physiological characteristics of the participant**

(l min^-1^ )	2.16
Relative (ml min^-1^ kg^-1^ BM)	31.5
Relative (ml min^-1^ kg^-1^ LM)	47.2
Maximal aerobic speed (km h^-1^)	8.58
HR_peak_ (beats min^-1^)	140
(l min^-1^)	102.4
CMJ P_peak_ (kW)	1.69
Relative CMJ P_peak_ (W kg^-1^)	25.2
SJ P_peak_ (kW)	1.46
Relative SJ P_peak_ (W kg^-1^)	21.7
MIVT left leg (Nm)	118.1
MIVT right leg (Nm)	109.4

BM, body mass; CMJ, countermovement jump; HR, heart rate; LM, lean mass; MIVT, maximal isometric voluntary torque; P_peak_, peak power; SJ, squat jump;  peak ventilatory expiration volume.

### Body composition

Total body mass and total lean mass were not altered during the marathon, while total fat mass was increased from pre-testing to the post marathon measurement (Table 2). The increase in fat mass was attributed to a large extent (97.1%) to a significant gain in fat mass in the core region. In the legs, total, lean, and fat masses were slightly but non-significantly reduced. The decrease in lean mass in the legs was based on decreases in the upper legs, while lean mass in the lower legs remained constant. Muscle density in the lower legs was non-significantly decreased after the marathon (Table 2).

**Table 2:** **Body composition before and after the marathon**

	Pre	Post	Change
Total body mass (kg)	68.2	68.8	+1.0%
Total lean mass (kg)	45.8	45.8	-0.0%
Total fat mass (kg)	19.4	20.0	+3.4%*
Total mass core (kg)	36.1	36.8	+2.0%
Lean mass core (kg)	22.4	22.4	+0.2%
Fat mass core (kg)	12.8	13.5	+5.2%*
Total mass legs (kg)	20.5	20.2	-1.6%
Lean mass legs (kg)	15.0	14.8	-1.1%
Fat mass legs (kg)	4.29	4.13	-3.7%
Lean mass upper leg (kg)	9.39	9.28	-1.2%
Lean mass lower leg (kg)	4.32	4.33	+0.2%
Muscle density lower leg (mg cm^-3^)	70.6	69.8	-1.2%

*, pre to post change >1.5 × coefficient of variation for this variable.

### Bone mineral content and density

Tibial vBMC and vBMD decreased significantly at the 4%-site in the right leg (Table 3). In the left leg, there was a significant increase in tibial vBMD at 4% of tibia length. Tibial vBMC decreased in the right leg at the 66%-site. At 14% and 38% of tibia length, there were no alterations in vBMC and vBMD from pre to post marathon (data not shown). At any position, no changes in the architecture of the bone were measured (Table 3). In the right leg, significant decreases in BMD at the wards and trochanter were present (Table 4). BMD at the upper left leg remained constant during the marathon. There was no difference in BMD in the lumbar spine from pre to post marathon (pre *vs.* post: 1.487 *vs.* 1.488 g∙cm^-2^).

**Table 3:** **Peripheral quantitative computed tomography derived values pre and post marathon at 4 and 66% of tibia length**

	Left			Right		
	Pre	Post	Change	Pre	Post	Change
vBMC_4%_ (g cm^-1^)	5.22	5.28	1.1%	5.12	5.01	-2.2%*
vBMD.tb_4%_ (mg cm^-3^)	274	277	1.2%*	278	272	-1.9%*
vBMD.tot_4%_ (mg cm^-3^)	334	336	0.4%	328	327	-0.3%
vBMC.tib_66%_ (g cm^-1^)	4.71	4.71	0.0%	4.78	4.76	-0.4%*
vBMD.ct.tib_66%_ (mg cm^-3^)	1013	1012	0.0%	1031	1028	-0.2%
Peri_66%_ (mm)	104	105	0.3%	102	102	0.2%
Endo_66%_ (mm)	81.5	81.7	0.3%	76.5	76.7	0.3%
Thk.ct_66%_ (mm)	3.65	3.66	0.3%	4.02	4.03	0.1%

vBMC, volumetric bone mineral content; vBMD, volumetric bone mineral density; tb, trabecular; tot, total; tib, tibia; ct, cortical; Peri, periosteal circumference; Endo, endosteal circumference; Thk.ct, cortical thickness. *, pre to post change >1.5 × coefficient of variation for this variable.

**Table 4:** **Bone mineral density of the upper femur pre and post marathon**

	Left leg			Right leg		
	Pre	Post	Change	Pre	Post	Change
Neck (g cm^-2^)	0.778	0.784	0.8%	0.740	0.740	-0.1%
Upper neck (g cm^-2^)	0.612	0.615	0.5%	0.542	0.548	1.2%
Lower neck (g cm^-2^)	0.943	0.952	0.9%	0.937	0.929	-0.9%
Wards (g cm^-2^)	0.692	0.700	1.1%	0.613	0.594	-3.1%*
Trochanter (g cm^-2^)	0.975	0.989	1.4%	0.897	0.879	-2.1%*
Shaft (g cm^-2^)	1.145	1.145	0.1%	1.094	1.085	-0.8%
Total (g cm^-2^)	1.011	1.017	0.6%	0.953	0.943	-1.0%

*, pre to post change >1.5 × coefficient of variation for this variable.

### Neuromuscular function

Absolute and relative peak power as well as peak velocity during a CMJ were decreased after the marathon (Table 5). CMJ peak force decreased only in the left leg (-5.4%), while it remained constant in the right leg (+0.6%). Peak power during CMJ decreased by 20.4 and 13.8% in the left and right leg, respectively. Total impulse during CMJ remained constant (pre: 75.7 N∙m∙s *vs.* post: 76.1 N∙m∙s). There was a slight decrease in both negative impulses (I_1_ and I_3_) and the positive impulse (I_2_) from pre to post (I_1_: -29.7 *vs.* -5.1 N∙m∙s; I_2_: 141.2 *vs.* 109.4 N∙m∙s; I_3_: -35.8 *vs.* -28.3 N∙m∙s). The time interval for I_1_ decreased (pre: 0.31 s *vs.* post: 0.24 s) and remained constant for I_2_ (pre: 0.36 s *vs.* post: 0.34 s) and I_3_ (pre: 0.05 s *vs.* post: 0.06 s). Peak power, peak velocity, and peak force during a SJ remained unaffected by the marathon. During both two-legged jumping maneuvers, peak force was not altered after the marathon. Maximum force during m1LH was reduced only in the left leg, while there was a non-significant reduction in the right leg (Table 5). MIVT was significantly reduced in the left leg by 14.5% (pre: 118.1 N∙m *vs.* post: 101.0 N∙m) and was maintained in the right leg (pre: 109.4 N∙m *vs.* post: 114.9 N∙m). Before the marathon, C was 0.196 ml O_2_∙kg^-1^∙m^-1^ = 4.098 J∙kg^-1^∙m^-1^ and after the marathon, C was 0.197 ml O_2_∙kg^-1^∙m^-1^ = 4.125 J∙kg^-1^∙m^-1^.

**Table 5:** **Jumping force, jumping velocity, and jumping power before and after the marathon**

	Countermovement jump			Squat jump			m1LH left leg			m1LH right leg		
	Pre	Post	Change	Pre	Post	Change	Pre	Post	Change	Pre	Post	Change
Peak force												
Absolute value (10^3^∙N)	1.29	1.25	-3.2%	1.54	1.51	-2.0%	1.26	1.10	-12.2%*	1.33	1.27	-4.7%
As a multiple of body weight	1.95	1.86	-4.8%	1.23	1.25	1.2%	2.00	1.76	-12.0%*	2.09	2.04	-2.4%
Peak velocity (m∙s^-1^)	1.72	1.47	-14.7%*	1.90	1.87	-2.0%	-	-	-	-	-	-
Peak power												
Absolute value (10^3^∙W)	1.69	1.42	-16.5%*	1.46	1.44	-1.2%	-	-	-	-	-	-
Per kg body mass (W∙kg^-1^)	25.2	20.6	-18.2%*	21.7	21.1	-2.7%	-	-	-	-	-	-

m1LH, multiple one-legged hopping. *, pre to post change >1.5 × coefficient of variation for this variable.

## Discussion

This is the first study investigating the physiological characteristics and alterations in body composition and neuromuscular function before and after the official successful completion of a marathon race in a nonagenarian man. We found that total fat mass increased in the final preparation phase before the race and that lean mass in the legs was slightly reduced after the marathon. Neuromuscular function declined during CMJ, while it remained constant during SJ. *F*_m1LH_ and MIVT were impaired after the marathon, but only in the left leg. Conversely, there were site-specific reductions in BMC and BMD in the lower tibia and upper femur in the right leg only.

 in our athlete was slightly lower than the one reported for life-long trained octogenarian athletes (2.56 l min^-1^ = 38 ml min^-1^ kg^-1^ body mass = 52 ml min^-1^ kg^-1^ lean mass; Trappe *et al*. 2013). This difference might be explained by the late start (at 64 years of age) with physical exercise in our participant. Nevertheless,  of our participant fits almost perfectly the regression line for  of endurance-trained men aged 50 to 81 years (expected  for a 90-years-old male = 33.3 ml min^-1^ kg^-1^ body mass; Kusy and Zielinski 2014). The reduced  of our participant is explained by the markedly lower peak heart rate (140 beats min^-1^) than the reported average peak heart rate in trained octogenarian athletes (160 beats min^-1^; Trappe *et al*. 2013). The reduced peak heart rate in the master athlete is independent of activity level, namely because of a reduced intrinsic heart rate and reduced chronotropic responsiveness to *β*-adrenergic stimulation (Christou and Seals 2008). In addition, the decrease in  might rely on an increased peripheral resistance due to a reduced elasticity of the arteries (Saltin 1986). The reduced  in our participant could also be due to the relatively low training volume and intensity associated with his training routine. In fact, the training routine of our participant consisted of only 3 h training per week on three separate days of the week, whereby two training sessions were comprised of low intensity training (~75% peak heart rate), while the intensity of the third training session was moderate (~85% peak heart rate).

In contrast to ,  was ~25% higher in our participant relative to the values reported for trained octogenarians (102 *vs.* 79 l min^-1^; Trappe *et al.*^2013^). It is assumed that a reduction in  in the aging process is mainly caused by a reduction of elastic recoil of the lung tissue and chest wall (Miller 2010). Given that  was markedly higher than expected we assume that lung tissue and chest wall elasticity were well maintained in our participant. Maximal muscular power during CMJ was markedly higher (25.2 W kg^-1^ body mass) than the age-predicted value for "healthy" males (reference value = 21.6 W kg^-1^ body mass; Runge *et al.*^2004^). This was rather surprising, as previous studies have shown that maximal muscular power in master endurance athletes is not different compared to inactive elderly people (Grassi *et al.*^1991^, Michaelis *et al.*^2008^). However, Grassi *et al.* ( 1991) observed that there was a trend for a more pronounced decrease in maximal muscular power in untrained people after age 65 years compared to master endurance athletes, which might explain the higher maximal muscular power in our 90-year-old athlete compared to an age-matched inactive reference.

Race time for our athlete was ~230% higher than the actual marathon world record (Figure 1). As can be seen in Figure 1, race time increases by 40% between M80 and M85. In this study, we showed that the increase in race time between M85 and M90 is of similar magnitude (49%), despite the massive reduction in running speed after 25 km in our nonagenarian athlete. The reduction in running speed was accompanied by a decrease in  from 77 to 55% . The decreases in both running speed and relative *V*O_2_ point to emptying of intrinsic energy stores. This is seen in the pronounced reduction in fat mass in the legs after the marathon despite an increase in total body fat mass and in the concomitant decreases in lean mass and muscle density that might point to a loss of glycogen, as we previously described in Ironman triathletes (Mueller *et al.*^2013^).

Hydration was well managed during the marathon, as there was no difference in total body and lean mass during the marathon. This stands in contrast to young world-class marathon runners, who usually lose ~8.8% body mass mainly due to sweat-induced fluid losses (Beis *et al.*^2012^). This discrepancy in fluid balance between our athlete and young world-class runners could be explained by the different total times spent for fluid intake, *i.e.* less than 60 s *vs.* several min. It was surprising that fat mass increased significantly during the 5 d span between the pretests and the measurements after the race. The participant was advised to report to the laboratory for pre-testing well nourished, that is with filled glycogen stores, to simulate pre-race conditions. Consequently, the athlete ingested a carbohydrate-rich and high-energy diet to comply with the requirements. During pre-testing, only a small part of the stored glycogen was drawn on for energy supply. In the remaining 5 d before the race, the participant continued his carbohydrate-rich and high-energy diet with the goal to assure filled glycogen stores at the start of the race. The further provided nutrients during this period was stored as fat because the glycogen stores were already filled. Unfortunately, the participant did not report his nutritional feedings during the pre-competition phase. Hence, we cannot confirm our assumption. The observed increase in fat mass mainly in the core region is thereby a typical phenomenon for men (Shimokata *et al.*^1989^). As shown by Cahill *et al.* ( 2013), increasing caloric intake by 70% for 7 d can lead to a gain in fat mass (primarily in the trunk and android region) of ~1 kg in young participants.

The measured decreases in vBMC, vBMD, and BMD in the right leg might point to actual decreases in bone mass. However, under physiological conditions, changes in bone variables are detectable only after several months and hence, actual decreases after 5 d are unlikely to occur and/or cannot be detected with the current technology. Based on this fact, we assume that the measured decreases might be attributed to changes in water balance within bone. It is known that mechanical loading leads to fluid flow out of the bone and to a contrarywise inflow after termination of loading (Piekarski and Munro 1977). A reduced water content of the bone leads in turn to a decrease in measured BMD (Mohiuddin 2013). In consideration of the distinct movement pattern of the participant, *i.e.* asymmetric running with a pronounced loading of the left leg and sparing of the right, we assume that fluid out- and inflow were different, with respect to body side and bone site. To our knowledge, only one study did measure vBMC in the lower leg directly before and after an extreme endurance event, showing no alteration at any site after an Ironman triathlon in recreationally active males (Mueller *et al.*^2013^).

*F*_m1LH_ and MIVT were remarkably different between the two legs. In the left leg, which was the leg affected by foot pain during the preparation phase, significant reductions were present in both variables. In addition, decreases in peak power during CMJ were pronounced in the left leg. The first point implies that a marathon did impair maximal voluntary ground reaction force in this leg. However, referring to the functional muscle-bone unit in the lower leg in a healthy state (Anliker and Toigo 2012), the bone mass at 14% of tibia length in our athlete (3.05 g∙cm^-1^) should be associated with a *F*_m1LH_ twice as high as actually measured (actual: 1.26 kN *vs.* reference: 2.55 kN). We assume that this discrepancy was mainly due to coordination problems during the jumping maneuver, which were aggravated by the marathon. Our finding that MIVT was reduced lends further credence to the findings of Nicol *et al.* ( 1991) and Petersen *et al.* ( 2007), who showed that experienced young marathon runners and highly-trained male runners, respectively, displayed a decrease of ~22% in MIVT after a marathon. Interestingly, no significant alterations in these variables were detected in the right leg. The distinct running pattern of our athlete might be an explanation for the side-differences in *F*_m1LH_, MIVT, and peak power during CMJ. He tended to tilt to the left side during running. The load on both legs was thereby disparate, which might have resulted in different fatigue responses in the two legs. Especially, the lack of loading during training in response to the left foot injury might have aggravated fatigue in this leg. As MIVT and *F*_m1LH_ were only reduced in the left leg and could be maintained in the right leg, the reduction in MIVT and *F*_m1LH_ in the left leg most likely was the result of peripheral fatigue and/or coordination problems. After long endurance exercise, peripheral fatigue might be attributed to reduced excitability of the sarcolemma (Millet *et al.*^2002^) or due to impaired sarcoplasmatic calcium release (Allen *et al.*^1995^).

The non-alteration in peak jumping force during the two-legged jumping maneuvers can be explained by the submaximal nature of these maneuvers. Peak force as a multiple of body weight per leg during CMJ was half the relative peak force during m1LH. Thus, our results implicates that the submaximal force measured during CMJ can be maintained even under fatigued conditions. Accordingly, total impulse also remained constant. Although the sum of all impulses remained constant, I_2_ (positive impulse) as well as I_1_ and I_3_ (negative impulses) were reduced. A clearly reduced countermovement magnitude (data not shown) led to a shorter and smaller I_1_ and caused thereby the less steep rise of the force curve. Decreases in peak power and peak velocity could be detected during CMJ but not SJ. This specificity could be attributed to the different power production modes associated with the two distinct jumping maneuvers (stretch-shortening cycle *vs.* shortening muscle action). Therefore, we assume that the type of power production during the competition was responsible for the fatigue after the marathon, while the other type of power production was not influenced. Our finding coincides with the results of Petersen *et al.* ( 2007), who showed in highly trained runners that mean concentric power during CMJ was reduced after fatiguing exercise.

*C* was not altered during the marathon in our participant. This finding was unexpected because the participant suffered from coordination problems during the second treadmill test, which in turn should increase *C*. In fact, it was shown that in younger recreationally active runners *C* was increased after exhausting exercise (Lazzer *et al.*^2014^). The unique running pattern of our participant, which is certainly different than the normal or conventional running technique, might represent a very efficient running pattern that is not influenced by fatigue. Despite the participant’s unusual running technique, *C* was not different when compared to the respective value found in recreationally active runners aged 17–54 years (Di Prampero *et al.*^1986^, Lazzer *et al.*^2014^). Accordingly, it was proposed that *C* does not depend on running experience and training in younger people (Slawinski and Billat 2004).

## Conclusions

In conclusion, this was the first study that investigated physiological characteristics of a nonagenarian runner before and after his first official marathon. Furthermore, it is the first report about the alterations in body composition and neuromuscular function found after a marathon in a nonagenarian athlete. Similarities in alterations in the measured parameters between young marathon runners (values from the published literature) and the nonagenarian athlete existed in leg composition, two-legged jumping performance, and BMD of the lumbar spine. In contrast, alterations in total body composition, BMC of the lower leg, BMD of the upper femur, and m1LH performance were markedly different in the nonagenarian athlete when compared to his younger counterparts. Measured decreases in BMC and BMD in the legs might be associated with changes in water balance within bone. Furthermore, there were significant side differences in *F*_m1LH_, MIVT, BMC, and BMD, which are attributed to the distinct non-symmetrical running pattern of the athlete. Potential challenges at the beginning of the marathon (*e.g.* coordination problems) were amplified during the competition. However, successful completion of a marathon is still feasible in a nonagenarian man. In consideration of the findings of the present study, there is room for improvement in a future competition of this athlete.
